# The Risk of Endoscopic Mucosal Resection in the Setting of Clopidogrel Use

**DOI:** 10.1155/2014/494157

**Published:** 2014-04-27

**Authors:** Vikneswaran Namasivayam, Ganapathy A. Prasad, Lori S. Lutzke, Kelly T. Dunagan, Lynn S. Borkenhagen, Ngozi I. Okoro, Yutaka Tomizawa, Navtej S. Buttar, Wongkeesong Louis Michel, Kenneth K. Wang

**Affiliations:** ^1^Department of Gastroenterology and Hepatology, Singapore General Hospital, Outram Road, Singapore 169608; ^2^Mayo Clinic, Division of Gastroenterology and Hepatology, 200 2nd Street SW, Rochester, MN 55905, USA

## Abstract

*Objective*. Guidelines on antiplatelet medication use during endoscopy are based on limited evidence. We investigate the risk of bleeding and ischemic events in patients undergoing endoscopic mucosal resection (EMR) of esophageal lesions in the setting of scheduled cessation and prompt resumption of clopidogrel. *Design*. Single centre retrospective review. *Patients*. Patients undergoing EMR of esophageal lesions. *Interventions*. Use of clopidogrel before EMR and resumption after EMR. Patients cease antiplatelets and anticoagulants 7 days before EMR and resume clopidogrel 2 days after EMR in average risk patients. *Main Outcomes*. Gastrointestinal bleeding (GIB) and ischemic events (IE) within 30 days of EMR. *Results*. 798 patients underwent 1716 EMR. 776 EMR were performed on patients on at least 1 antiplatelet/anticoagulant (APAC). 17 EMR were performed following clopidogrel cessation. There were 14 GIB and 2 IE. GIB risk in the setting of recent clopidogrel alone (0%) was comparable to those not on APAC (1.1%) (*P* = 1.0). IE risk on clopidogrel (6.3%) was higher than those not on APAC (0.1%) (*P* = 0.03). *Limitations*. Retrospective study. *Conclusions*. Temporary cessation of clopidogrel before EMR and prompt resumption is not associated with an increased risk of gastrointestinal bleeding but may be associated with increased ischemic events.

## 1. Background and Aims

Endoscopic mucosal resection (EMR) has emerged as an alternative to esophagectomy in the treatment of high grade dysplasia (HGD) and intramucosal esophageal carcinoma (EC) [1]. It involves the resection of a localized segment of diseased mucosa which results in an ulcer. It incurs a higher risk of complications than a diagnostic endoscopy, including gastrointestinal bleeding in 13–17% of patients [2, 3].

EMR is often performed in elderly patients [2] with ischemic comorbidities [3] that require the use of antiplatelet agents such as clopidogrel. Clopidogrel is used either in combination or as an alternative to aspirin in acute coronary syndrome and it reduces the risk of ischemic stroke, myocardial infarction, or vascular death [4, 5]. Cardiology guidelines recommend a minimum of 12 months of dual antiplatelet therapy after implantation of drug-eluting stents [6, 7] to mitigate the risk of stent thrombosis. However, dual antiplatelet therapy incurs a 4% risk of bleeding [8] mainly from the gastrointestinal tract with its attendant morbidity and mortality. Observational studies also suggest that concomitant use of clopidogrel and proton pump inhibitors (PPI) may attenuate clopidogrel's efficacy resulting in increased cardiovascular events [9, 10]. This may be relevant in patients with Barrett's esophagus undergoing EMR who may also be on PPI therapy for reflux. Patients who are on clopidogrel-based antiplatelet therapy prior to EMR represent a subgroup that may be at an increased risk of bleeding or ischemic events. However, there is a paucity of evidence to guide decision making regarding the management of antiplatelet use in the setting of EMR [11].

This retrospective cohort study assesses the risk of bleeding and ischemic events following EMR in a tertiary referral centre that routinely institutes temporary cessation of clopidogrel before and after EMR. EMR was chosen since these were iatrogenic ulcers of uniform depth and size all created in a similar organ, the esophagus.

## 2. Methods

### 2.1. Study Design

Data from a prospectively maintained database of consecutive patients with HGD and EC undergoing endoscopic treatment between 1994 and 2009 at the Barrett's Esophagus Unit at the Mayo Clinic (Rochester, MN) was reviewed. All patients underwent 4-quadrant biopsies for every centimeter of visible BE segment. Baseline assessments in all patients include endoscopic ultrasound and computerized tomography scans of the chest and upper abdomen. Positron emission tomography scans to exclude distant metastases were performed since 2003 if there was evidence of carcinoma.

### 2.2. EMR

EMR was performed as previously described [12] as an ambulatory procedure. The initial technique involved ligation with a Bard Six-Shooter (Bard Interventional Products, Billerica, MA). From April 2000, EMR was performed using a cap (EMR-001, Olympus America Inc., Center Valley, PA). Since 2004, EMR also was performed using the Duette multiband mucosectomy device (Cook Ireland, Limerick, Ireland) as previously described [13].

### 2.3. Pre-EMR and Follow-Up Evaluation

All patients were assessed prior to endoscopy by the physicians, nurse practitioner, or clinical coordinators. Information on baseline health status as well as current and past medication use including antiplatelets, NSAIDs, and anticoagulants was obtained. Patients were advised to discontinue clopidogrel 7 days prior to EMR and this was verified before each endoscopy. In patients with high-risk cardiac conditions, clopidogrel was discontinued after discussion with the cardiologist. In patients on warfarin, an INR below 1.5 was used to determine ability to proceed with EMR.

All patients were placed on twice-daily PPI therapy after EMR at the standard doses. Patients were educated carefully regarding EMR and their possible immediate and delayed complications by the physicians, nurse practitioner, and clinical coordinators. Patients were advised to avoid the use of clopidogrel for 2 days and aspirin and NSAIDs for 2 weeks after EMR. Patients were contacted by phone within 2–5 days of their procedures to determine their status. Follow-up evaluation included endoscopic surveillance with biopsies and EMR if indicated, performed every 3 months for 2 years, then every 6 months for 1 to 2 years, and annually thereafter. Data on complications rates including bleeding was collected prospectively. In patients requiring inpatient treatment in another hospital in between EMR sessions, medical records were routinely obtained to update the database.

### 2.4. Statistical Analysis

Data management and statistical analysis were performed using JMP software (version 8.0, SAS Institute Inc., Cary, NC). Baseline continuous data were compared using the 2-sample *t*-test or Wilcoxon rank-sum test depending on the data normality. Baseline categorical data were compared using the chi-squared test and Fisher's exact test when the sample size was small.

### 2.5. Risk Assessment

Gastrointestinal bleeding (GIB) was defined as bleeding requiring hospitalization, blood transfusion, endoscopic intervention, or surgery or bleeding associated with a more than 2 g drop in hemoglobin within 30 days of EMR. The risk of GIB is defined as the percentage of total number of EMR resulting in GIB. Ischemic events (IE) were defined as cardiovascular mortality, thrombotic stroke, myocardial infarction, hospitalization for cardiac or cerebrovascular causes, cardiac catheterization, or coronary bypass for acute coronary syndrome within 30 days of EMR. A prospective EMR database was cross-referenced with electronic medical records to ascertain use of antiplatelets, NSAIDs, and anticoagulants and episodes of GIB and IE following EMR. The relative risk of GIB was defined as the ratio of GIB in patients on clopidogrel and GIB in patients not on APAC. The relative risk of IE was defined as the ratio of IE in patients on clopidogrel and patients not on APAC. This study was approved by the Institutional Review Board.

## 3. Results

798 patients (654 males) underwent 1716 sessions of EMR. Median age was 69 years. 8 patients who had undergone 18 EMR with missing data on prior APAC use were excluded from further analysis. There were a total of 1698 sessions of EMR included for further analysis. Each patient underwent between 1 and 18 EMR.

There were a total 776 EMR performed in the setting of recent APAC use and 922 on patients who were not on APAC (Table 1). Of those performed on patients with recent APAC use, 595 (76.7%) had APAC discontinued before the EMR, 26 EMR (3.4%) were performed on APAC, 3 (0.4%) patients were bridged on low molecular weight heparin, and data was missing on 152 EMR (19.6%).

**Figure 1:**
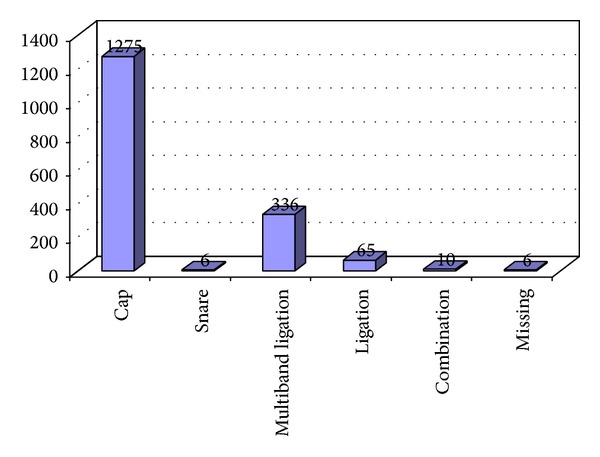
Techniques of EMR used.

1555 EMR (91.6%) were performed on PPI, 4 were on H2 blockers (0.2%), 1 was on a combination of PPI and H2 blocker (0.1%), 135 were performed without any acid suppressive medication (8.0%), and there was missing information on 3 procedures (0.2%). Most (75%) of the EMR were performed using the Olympus EMR cap method (Figure 1).

### 3.1. GIB Risk (Table 2)

There were 14 (0.8%) cases of GIB following EMR. The risk of GIB on any APAC was 0.52% versus those never on APAC being 1.09% (*P* = 0.28). The risk of GIB in the setting of recent clopidogrel alone was 0% versus those never on APAC being 1.09% (*P* = 1.0). The likelihood of GIB from EMR performed while on PPI was similar to that performed without any acid blocking medication (0.84% versus 0.74%, *P* = 1.0). There was no statistically significant difference in risk of GIB in EMR performed with missing information on APAC discontinuation prior to EMR versus those with documented history on discontinuation of APAC (0% versus 0.64%, *P* = 1.0).

97 of the 1698 EMR involved insertion of hemostatic clips. Clip insertion was more likely in those performed in the setting of recent APAC use (7.7%) than those not on APAC to begin with (4.1%) (*P* < 0.005). The likelihood of GIB in EMR involving clip insertion was not statistically different from EMR not requiring clip insertion (1.03% versus 0.75%, *P* = 0.54).

### 3.2. IE Risk (Table 2)

There were 2 IE in total. One patient had ST elevation myocardial infarction on the day of EMR and the other had a sudden cardiac death 28 days after EMR. The risk of IE on any APAC was 0.13% versus those not on APAC being 0.11% (*P* = 1.0). The risk of IE in the setting of recent clopidogrel alone was 6.3% versus those not on any APAC being 0.1% (*P* < 0.05).

The risk of IE in patients with preexisting ischemic heart disease was not significantly different from those without ischemic heart disease (0.25% versus 0.08%, *P* = 0.43). The risk of IE from EMR performed on PPI was similar to that performed off PPI (0.07% versus 0.76%, *P* = 0.15). All patients on clopidogrel in this study were also on PPI. There was no significant difference in risk of IE in EMR performed with missing information on APAC discontinuation prior to EMR versus those with documented history on discontinuation of APAC (0.13% versus 0.16%, *P* = 1.0). There was no significant difference in IE (*P* = 0.67) and GIB (*P* = 0.16) rates for the various EMR techniques.

## 4. Discussion

In this large retrospective cohort study, there was no significant difference in gastrointestinal bleeding following EMR between those on clopidogrel who had a scheduled cessation and those not on any antiplatelet therapy. The risk of ischemic events appears to be significantly higher following EMR in patients on clopidogrel who had a scheduled cessation compared to those who were not on any antiplatelet therapy before EMR.

There is a paucity of evidence-based algorithms to guide the management of antithrombotic therapy in patients undergoing EMR. Expert guidelines recommending cessation of clopidogrel 7–10 days prior to high risk endoscopy are based on low quality evidence (i.e., further research is very likely to have an important impact on confidence in the estimate of effect and is likely to change the estimate) [11]. Case series on EMR have consistently reported a significant risk of bleeding of 4–12% [2, 3]. However, patients undergoing EMR are usually elderly with cardiac comorbidities which necessitate antiplatelet therapy. An earlier study from the authors' centre reported 38% prevalence of cardiac disease in patients undergoing esophageal EMR [3].

We have demonstrated that the bleeding risk with EMR is not increased with resumption of clopidogrel two days after EMR, following a scheduled protocol of temporary cessation prior to EMR. It is uncertain whether clopidogrel causes mucosal injury. It may cause rebleeding due to impaired hemostasis in patients with underlying mucosal defects [14].

We assessed for possible confounding by clip insertion. Prophylactic clip insertion was more likely during EMR in patients on APAC suggesting a potential bias that may account for the low overall rate of bleeding in our series. However this did not contribute to a difference in bleeding rates. Acid blockade with PPI as well as differences in EMR techniques did not result in a difference in bleeding rates.

The risk of ischemic events appears to be significantly increased with cessation of clopidogrel prior to EMR. This may be a reflection of the underlying cardiac comorbidity present in this elderly population. However, the risk of ischemic events was not significantly different between those with and without ischemic heart disease. The low rate of events precludes multivariate analysis for possible confounding by preexisting heart disease and other risk factors for vascular disease. This needs to be clarified in larger cohort studies. The absolute number of ischemic events and the total number of EMR performed on clopidogrel is small and hence it is possible that a slight change in the number of ischemic events may have altered the magnitude of risk as it is evident from the wide confidence interval. The endpoint of ischemic events (IE) was defined as a composite of clinically relevant hard endpoints to mitigate any bias. The 30-day window was chosen as an arbitrary time window within which the effects of any change in antiplatelet therapy were likely to manifest.

Of note, 152 of the 1698 EMR had missing data on whether antiplatelet therapy was actually discontinued prior to EMR as advised. This data was included in our analysis to reflect actual clinical practice. There was no difference in the bleeding and ischemic outcomes in patients with complete and missing data on cessation of antiplatelet therapy.

This study has several limitations. There was no prospective confirmation of continued antiplatelet cessation following EMR for the scheduled duration. Hence it is possible that patients may have resumed antiplatelet therapy later than advised. This study does not provide the actual risk of bleeding with clopidogrel as it is discontinued prior to EMR. However, a randomized study assessing the risk of complications with continued clopidogrel therapy is probably not feasible and perhaps unethical. It is noteworthy that there were no bleeding events despite resumption of clopidogrel within 48 hours. This would suggest that clopidogrel may be safely resumed within 2 days without the need for a protracted period of cessation following EMR.

In this series, patients underwent between 1 and 18 EMR. The repeated endoscopic evaluation and clinical review provide an opportunity to record clinically significant complications occurring after the EMR. Data on antiplatelets, NSAIDs, and anticoagulant use was recorded prospectively thereby minimising recall bias.

In conclusion, prompt resumption of clopidogrel following temporary cessation for EMR is not associated with an increased risk of gastrointestinal bleeding. The temporary cessation of clopidogrel may be associated with an increased risk of ischemic events which may be related to the underlying cardiac comorbidities of the patient population and needs to be confirmed in prospective studies.

## Figures and Tables

**Table 1:** Antiplatelet/anticoagulant use.

Antiplatelet/anticoagulant (APAC)	Total number of EMR performed	Frequency of GIB	Frequency of IE
None	922	10	1
Coumadin	89	0	0
Clopidogrel	17	0	1
Aspirin	467	1	0
NSAID	44	0	0
Coumadin and aspirin	63	0	0
Aspirin and NSAID	44	0	0
Coumadin, clopidogrel, and aspirin	3	1	0
Clopidogrel and aspirin	40	2	0
Coumadin and NSAID	1	0	0
Coumadin and clopidogrel	1	0	0
NSAID, clopidogrel, and aspirin	5	0	0
Coumadin, aspirin, and NSAID	2	0	0
Total	**1698**	**14**	**2**

**Table 2:** Comparison of outcomes in the two groups.

	Clopidogrel	No APAC
Total number of EMR (*N*)	17	922
GIB (*N*)	0	10
Risk of GIB	0%	1.1%
Relative risk of GIB	0	
IE	1	1
Risk of IE	6.3%	0.1%
Relative risk of IE	63 (CI 4–862).	

## References

[1] Wang KK, Sampliner RE (2008). Updated guidelines 2008 for the diagnosis, surveillance and therapy of Barrett’s esophagus. *American Journal of Gastroenterology*.

[2] Pech O, Behrens A, May A (2008). Long-term results and risk factor analysis for recurrence after curative endoscopic therapy in 349 patients with high-grade intraepithelial neoplasia and mucosal adenocarcinoma in Barrett’s oesophagus. *Gut*.

[3] Prasad GA, Wu TT, Wigle DA (2009). Endoscopic and Surgical Treatment of Mucosal (T1a) Esophageal Adenocarcinoma in Barrett’s Esophagus. *Gastroenterology*.

[4] Anderson JL, Adams CD, Antman EM (2007). ACC/AHA 2007 guidelines for the management of patients with unstable angina/non ST-elevation myocardial infarction: a report of the American College of Cardiology/American Heart Association Task Force on Practice Guidelines (Writing Committee to Revise the 2002 Guidelines for the Management of Patients With Unstable Angina/Non ST-Elevation Myocardial Infarction): developed in collaboration with. *Circulation*.

[5] Gent M (1996). A randomised, blinded, trial of clopidogrel versus aspirin in patients at risk of ischaemic events (CAPRIE). *The Lancet*.

[6] Grines CL, Bonow RO, Casey DE (2007). Prevention of premature discontinuation of dual antiplatelet therapy in patients with coronary artery stents: a science advisory from the American Heart Association, American College of Cardiology, Society for Cardiovascular Angiography and Interventions, American College of Surgeons, and American Dental Association, with representation from the American College of Physicians. *Circulation*.

[7] Hodgson JM, Stone GW, Lincoff AM (2007). Late stent thrombosis: considerations and practical advice for the use of drug-eluting stents: a report from the Society for Cardiovascular Angiography and Interventions Drug-Eluting Stent Task Force. *Catheterization and Cardiovascular Interventions*.

[8] Yusuf S, Zhao F, Mehta SR, Chrolavicius S, Tognoni G, Fox KK (2001). Effects of clopidogrel in addition to aspirin in patients with acute coronary syndromes without ST-segment elevation. *The New England Journal of Medicine*.

[9] Ho PM, Maddox TM, Wang L (2009). Risk of adverse outcomes associated with concomitant use of clopidogrel and proton pump inhibitors following acute coronary syndrome. *Journal of the American Medical Association*.

[10] Juurlink DN, Gomes T, Ko DT (2009). A population-based study of the drug interaction between proton pump inhibitors and clopidogrel. *Canadian Medical Association Journal*.

[11] Anderson MA, Ben-Menachem T, Gan SI (2009). Management of antithrombotic agents for endoscopic procedures. *Gastrointestinal Endoscopy*.

[12] Prasad GA, Buttar NS, Wongkeesong LM (2007). Significance of neoplastic involvement of margins obtained by endoscopic mucosal resection in Barrett’s esophagus. *American Journal of Gastroenterology*.

[13] Peters FP, Kara MA, Curvers WL (2007). Multiband mucosectomy for endoscopic resection of Barrett’s esophagus: feasibility study with matched historical controls. *European Journal of Gastroenterology and Hepatology*.

[14] Ng FH, Wong SY, Chang CM (2003). High incidence of clopidogrel-associated gastrointestinal bleeding in patients with previous peptic ulcer disease. *Alimentary Pharmacology and Therapeutics*.

